# Fabrication of Chitosan/Konjac Glucomannan Active Composite Films Loaded with Ethyl-Cellulose-Coated D-Limonene Nanoparticles for Fresh Beef Preservation

**DOI:** 10.3390/foods15213874

**Published:** 2026-10-29

**Authors:** Fangling Wu, Lingli Chen, Chuanlong Yu, Hui Chen, Yi Han, Wenbing Hu, Suyun Lin, Xiong Li, Chunlan Liu, Kehui Ouyang, Wenjun Wang

**Affiliations:** 1 College of Food Science and Engineering, Jiangxi Agricultural University, Nanchang 330045, China; wfling10102022@163.com (F.W.); chenlingli89@163.com (L.C.); yuchuanlongvip@163.com (C.Y.); ch2568@jxau.edu.cn (H.C.); hy1087@jxau.edu.cn (Y.H.); hu_wenbing@163.com (W.H.); ncusklinsuyun@163.com (S.L.); 2 Pingxiang Center for Agricultural Science and Technology Research, Pingxiang 337109, China; lixiong1998@163.com; 3 Slong Beef Industry Co., Ltd., Pingxiang 337109, China; chunlanliu011@163.com; 4 College of Animal Science and Technology, Jiangxi Agricultural University, Nanchang 330045, China

**Keywords:** chitosan, konjac glucomannan, D-limonene, edible film, food packaging material

## Abstract

Petroleum-derived plastic packaging suffers from poor degradability, environmental pollution and small-molecule migration. To develop high-performance green active packaging, we fabricated composite films via solution casting using chitosan (CS), konjac glucomannan (KGM), and ethyl-cellulose-coated D-limonene nanoparticles (DENPs). Compared with the CS/KGM control film, the optimized CS/KGM/DENPs 15% film increased tensile strength by 72% (18.05 MPa) and elongation at break by 67% (46.65%), and achieved improved UV shielding, water vapor and oxygen barrier performance, with oxygen permeability decreased to 5.32 × 10^−8^ g·mm/m^2^·s·Pa. Fourier-transform infrared spectroscopy (FTIR) confirmed intermolecular hydrogen bond interactions between CS and KGM. The composite films exerted obvious antibacterial activity against *Escherichia coli*, *Pseudomonas aeruginosa*, *Staphylococcus aureus*, and *Brochothrix thermosphacta*. When applied for chilled-beef preservation, the CS/KGM/DENPs 15% film effectively retarded beef spoilage, maintained meat color and pH, and suppressed lipid–protein oxidation. Under the present experimental conditions, it prolonged the acceptable storage period of chilled beef to approximately 15 d. Meanwhile, the composite film achieved a maximum weight loss of 85.66% within 28 d during soil burial disintegration, showing great potential as an eco-friendly active food-packaging material for industrial applications.

## 1. Introduction

Beef is a high-protein ingredient abundant in moisture and nutrients, yet it easily undergoes spoilage when preserved [1]. Consequently, developing strategies to delay beef deterioration and lengthening its shelf life has become an urgent task. Refrigeration and freezing serve as fundamental storage modes for beef preservation. Freezing tends to trigger juice exudation and loss of nutritional components, while refrigeration achieves superior fresh-keeping performance, albeit with a relatively short shelf life, and it is commonly used in combination with plastic wrap. To further extend preservation duration, traditional beef preservation strategies combine such low-temperature storage with chemical preservatives or ordinary plastic packaging. Conventional plastic packaging merely functions as a physical barrier and can indirectly slow microbial reproduction, but it provides no intrinsic antibacterial effect and can hardly curb microorganism-induced spoilage effectively [2]. To overcome the drawbacks of conventional packaging, antibacterial-active packaging has emerged accordingly. Nevertheless, many existing products suffer unsatisfactory practical performance, such as unstable antibacterial efficiency or poor material compatibility [3]. Therefore, developing packaging materials that are biodegradable, antibacterial, and have good mechanical properties has become an important way to solve the problem of beef preservation and ensure food safety.

In recent years, antibacterial packaging materials were extensively adopted to curb the deterioration of chilled fresh meat. Lipids, polysaccharides and proteins served as typical substrates to produce biodegradable antibacterial film [4]. Among them, due to their wide availability, good film-forming ability, biocompatibility, and remarkable antibacterial and barrier properties, polysaccharide materials present broad application prospects in active preservation packaging. Chitosan (CS) displayed favorable application prospects in degradable active composite films due to its intrinsic antibacterial trait, whereas pure CS films suffered insufficient water resistance and mechanical properties [5,6]. Konjac glucomannan (KGM) has good film-forming ability and biocompatibility. When blended with chitosan, the resultant composite possesses favorable water resistance and reported disintegration, and can act as a stable carrier for active compounds [7].

Plant essential oils, as widely used natural volatile substances in food packaging, possess significant antibacterial activity [8]. D-limonene, as a natural plant extract, exhibits excellent antibacterial and antioxidant properties [9]. When loaded onto a composite film, it significantly enhances the functional characteristics of the film. Previous studies have found that directly adding D-limonene to the composite film improved the electrostatic interactions, hydrogen bonds, and hydrophobic interactions between the film components, and enhanced antioxidant and antibacterial properties [10,11]. However, D-limonene has poor stability and is prone to volatilization and oxidation decomposition. It is difficult to achieve long-term effects through direct addition and it may affect the appearance and performance of the film. To improve the stability and adaptability of essential oils, technologies such as nanoemulsions, Pickering emulsions, and nanoparticle encapsulation have been developed successively [12,13]. Although nanoemulsions can enhance the dispersion of essential oils, they have high interfacial tension and are not conducive to long-term storage [14]; Pickering emulsions have stronger stability, but their particle size distribution is wider and their controlled-release ability is limited, making it difficult to precisely control the release rate of essential oils [15]. In contrast, nanoparticle encapsulation technology can precisely encapsulate essential oils through the coating material, significantly improving the stability of essential oils, reducing volatilization and oxidation losses, achieving controlled release of active components, and being well compatible with film solutions, improving the stability performance of the composite film [16].

Our previous research [17] successfully fabricated and optimized D-limonene-loaded ethyl cellulose nanoparticles (DENPs), focusing on the optimization of encapsulation parameters for pure DENPs, but did not explore the interfacial interactions between DENPs and polysaccharide film matrix, nor did we conduct evaluations on meat preservation and soil-burial disintegration of composite films. Building upon the optimized DENPs from our prior work, the present study incorporates DENPs into the CS/KGM film matrix. This nano-encapsulation strategy effectively improves the compatibility of D-limonene within the CS/KGM film matrix, reduces the volatilization of antibacterial active components, and enhances comprehensive properties including mechanical strength and flexibility of the composite films. The mechanical properties, barrier performance, thermal stability, antibacterial activity, and soil-burial weight loss and disintegration performance of composite films with different nanoparticle dosages were systematically characterized. The composite film with optimal comprehensive performance was further applied to chilled-beef preservation to investigate its regulatory effects on meat freshness quality during cold storage. This study provides a feasible technical reference for developing efficient, environmentally friendly and multifunctional active packaging films for fresh meat preservation.

## 2. Materials and Methods

### 2.1. Materials and Chemical Reagents

Chitosan (molecular weight ≈ 300 kDa, degree of deacetylation (DDA) ≥ 85%) was obtained from Macklin Biochemical Co., Ltd., Shanghai, China. Konjac glucomannan (purity > 85%, viscosity > 15,000 mPa·s) was purchased from Hefei BASF Biotechnology Co., Ltd., Hefei, China. D-limonene (purity ≥ 95%, extracted from citrus) was supplied by Shanghai Yien Chemical Technology Co., Ltd., Shanghai, China. Ethyl cellulose (ethoxyl content: 48–49.5%, degree of substitution (DS): 2.2~2.5) was purchased from Shanghai Aladdin Biochemical Technology Co., Ltd., Shanghai, China. All chemicals used in this study were of analytical grade.

### 2.2. Preparation of Nanoparticles

The preparation of ethyl-cellulose-encapsulated D-limonene nanoparticles (DENPs) was performed according to our previously published method [17]. Briefly, 0.85 g EC powder together with 0.77 mL D-limonene was dispersed in 20 mL anhydrous ethanol under continuous stirring at 600 r/min until full solvation, which yielded a uniform ethanol-based organic phase. At an organic phase–solvent ratio of 20:100, the ethanolic organic solution was dripped into 100 mL deionized water at 3 r/min with continuous stirring at room temperature (25 ± 2 °C). Constant agitation facilitated self-assembly between EC and D-limonene for the generation of the D-limonene nanoparticle suspension.

### 2.3. Preparation of Films

Fabrication of the CK-DENPs composite film followed the experimental protocol reported by Chen et al. [18] with partial adjustments. Briefly, 0.15 g KGM powder was scattered into 50 mL deionized water for the preparation of a 0.3% (*w*/*v*) KGM aqueous solution. After that, 0.3 g CS powder was accurately weighed and separately solvated with 50, 45, 40, 35 and 30 mL of deionized water. After adding 1 mL of glacial acetic acid to each solution, the mixtures were stirred at room temperature for 1 h. Finally, nanoparticle suspensions with diverse loading volumes (0, 5, 10, 15, 20 mL) were incorporated into the blended liquid of CS and KGM to produce a uniform mixed liquid. The addition amounts of nanoparticles were 0%, 5%, 10%, 15%, and 20%, respectively. A total of 30 mL of the mixed solution was poured onto 10 cm × 10 cm Petri dishes, followed by drying at 37 °C using the solution-casting technique. The obtained composite films were labeled CK, CK-DENPs 5%, CK-DENPs 10%, CK-DENPs 15% and CK-DENPs 20%, respectively.

### 2.4. Characterization of DENPs

The micro-morphology of DENP aqueous dispersion was observed by a scanning electron microscope (SEM, SU8010, Hitachi, Tokyo, Japan), and the samples were subjected to sputter gold-coating before measurement. A nanoparticle size analyzer (NanoBrook Omni, Brookhaven Instruments Corporation, Nashua, NY, USA) was used to determine the average particle size, polydispersity index (PDI) and zeta-potential. Ten milliliters of freshly prepared DENP dispersion was heated in a water bath at 40, 55, 70, 85 and 100 °C for 60 min. After cooling to room temperature, the retention rate of D-limonene was measured. Thirty milliliters of freshly prepared DENP dispersion was stored at 4 °C for 16 d. Samples were collected every 2 d to determine the retention rate of D-limonene.

### 2.5. Structural and Functional Tests of Film

#### 2.5.1. Structural and Surface Property Characterization of Composite Films

Green labels were placed on films to shoot their digital images. For scanning electron microscopy observation (ZEISS Sigma 300, ZEISS, Oberkochen, Germany), cross-sectional specimens were first prepared by fracturing composite films in liquid nitrogen. The surface and cross-section microstructures of composite films were then characterized using this instrument.

The chromaticity of the film was measured using the Color Quest XE (Hunterlab, Reston, VA, USA) for its luminance (L*), redness (a*), and yellowness (b*). A standard white board (L_0_ = 94.62, a_0_ = −0.9, b_0_ = 0.59) was used as a reference. The total chromaticity difference (ΔE), whiteness index (WI), and yellowness index (YI) were calculated using Equations (1)–(3).

A Fourier-transform infrared spectrometer (Thermo Fisher Scientific, Waltham, MA, USA) was utilized to characterize the composite film’s chemical structure. The method proposed by Yan et al. [19] was adopted with minor modifications. Using air as the blank, transmittance signals spanning 400~4000 cm^−1^ were acquired at 4 cm^−1^ resolution via 32 replicate scans for every sample.

Transmittance of the composite films was determined using a UV–vis spectrophotometer (UV-5200PC, Yuanxi Instruments Co., Ltd., Shanghai, China) following the procedure of Yong et al. [20]. The film samples were cut into rectangles of 10 mm × 40 mm and placed into the quartz cuvette to closely attach to the inner wall of the cuvette. A blank quartz cuvette was used as the control. The scanning resolution was 0.5 nm, and measurements were conducted in triplicate over the wavelength range of 200–800 nm.

X-ray diffraction (XRD) analysis was performed using a Bruker D8 Venture diffractometer (Bruker, Karlsruhe, Germany) to characterize the crystal structure of the active packaging film. The operating voltage was set to 40 kV, the scan rate was 5°/min, and the scanning range was 5° to 90°.

The static water contact angle of the films was measured via the sessile drop method using a contact angle goniometer (OCA 20, Dataphysics, Filderstadt, Germany). A 3 μL droplet of deionized water was deposited on the film surface, and the contact angle value was recorded at 5 s after the droplet contacted the film surface. Five replicate measurements were performed at different positions on each film sample.

(1) $$∆E=\sqrt{(L^{*}-L_{0}{)}^{2}-(a^{*}-a_{0}{)}^{2}+(b^{*}-b_{0}{)}^{2}}$$

(2) $$WI=100-\sqrt{({100-L)}^{2}+a^{2}+b^{2}}$$

(3) $$YI=\frac{{142.86b}^{*}}{L^{*}}$$

where L_0_, a_0_, and b_0_ correspond to color metrics of standard white plate, and L*, a*, and b* refer to color metrics of the fabricated composite films.

#### 2.5.2. Thermal Stability of Composite Films

The thermal stability of composite films was analyzed using a simultaneous thermal analyzer (Netzsch TG 209 F3, Netzsch, Selb, Germany), following the experimental protocol reported by Lee et al. [21] with minor adjustments. Composite film specimens (10–20 mg) were loaded into an aluminum pan. Thermogravimetric (TG) and derivative thermogravimetric (DTG) curves of the samples were measured. Nitrogen was used as the inert shielding gas at a flow rate of 100 mL/min, and the samples were heated from 30 °C to 800 °C at a heating rate of 20 °C/min.

#### 2.5.3. Mechanical Properties Tests

A digital electronic micrometer was adopted to test film thickness, with a precision of 0.001 mm. Following the testing scheme proposed by Chen et al. [22], a texture analyzer (TA.XTplusC, Stable Micro Systems, Godalming, UK) manufactured by Stable Micro Systems was employed to quantify the film’s tensile strength (TS, MPa) and elongation at break (EAB, %). The composite films were cut into 10 mm × 60 mm rectangular strips. The initial fixture separation was 30 mm at a tensile rate of 1 mm/s. Mechanical properties were calculated via Equations (4) and (5).

(4) $$TS\left(MPa\right)=\frac{F}{d\times w}$$

(5) $$EAB\left(\%\right)=\frac{∆L}{L_{0}}$$

where F stands for the peak tensile load upon breakage (N), w represents the width of film specimens (mm), and d corresponds to film thickness (mm). ΔL indicates the stretching length at film fracture (mm), while L_0_ refers to the original separation of the two testing clamps (mm).

#### 2.5.4. Water Resistance and Barrier Performances Tests

The experimental scheme reported by Zhang et al. [23] was adopted with minor adjustments. The film was cut into squares (2 cm × 2 cm) and weighed (M_0_). Then, it was dried at 105 °C until a constant weight is achieved and then it was weighed again (M_1_). The dried film specimen was soaked in deionized water, kept under ambient temperature for 24 h, dried completely and then weighed to record mass M_2_. It was dried again at 105 °C until a constant weight was achieved and then weighed again (M_3_). Calculations were carried out according to Equations (6)–(8). The water vapor transmission property of each film was determined based on the experimental protocol from Fan et al. [24]. Briefly, 40 mL deionized water was transferred into centrifuge tubes. Each tube opening was covered with film, fastened by rubber rings and wrapped with sealing film, and then placed in a desiccator filled with anhydrous calcium chloride. After 12 h storage at 25 °C, the initial and final masses were recorded separately for calculation via Equation (9). Minor adjustments were made to the testing scheme from Fan et al. [24] for measuring the oxygen transmission coefficient (OP). A total of 3 g oxygen scavenger mixture (reduced iron powder:NaCl:activated carbon = 0.5:1.5:1) was added into centrifuge tubes. The tube mouth was covered with film before placing the tube into a desiccator with anhydrous silica gel. After 48 h incubation at 25 °C, initial and final masses were recorded and calculated by Equation (10).

(6) $$MC=\frac{{(M}_{0}-M_{1})}{M_{0}}\times 100$$

(7) $$WS=\frac{{(M}_{1}-M_{3})}{M_{1}}\times 100$$

(8) $$SD=\frac{M_{2}-M_{1}}{M_{1}}\times 100$$

(9) $$WVP=\frac{∆m\times d}{A\times t\times ∆p}$$

(10) $$OP=\frac{∆m\times d}{A\times t\times ∆p}$$

where Δm means mass fluctuation (g); d refers to sample thickness (m); A stands for the film-testing area (m_2_); t is the equilibrium period (s); and Δp represents the transmembrane partial pressure gradient between water vapor and oxygen (Pa).

#### 2.5.5. Antioxidant Activity of the Films

The methodology in this section was carried out based on the work of Wu et al. [25] with slight modifications. For the DPPH assay, DPPH powder was dissolved in anhydrous ethanol. Briefly, 0.0197 g of DPPH powder was dissolved and made up to 10 mL with anhydrous ethanol to prepare 5 mM DPPH stock solution. The stock solution was diluted 50-fold with anhydrous ethanol to obtain a 0.1 mM DPPH working solution. Then, 40 mg of composite film was added to 4 mL of the 0.1 mM DPPH working solution. For the ABTS assay, the ABTS working solution was prepared by mixing 7.4 mM ABTS stock solution and 2.6 mM potassium persulfate solution at a volume ratio of 1:1. The mixture was kept in the dark at room temperature for 16 h, and then diluted with deionized water to achieve an absorbance of 0.7 ± 0.02 at 734 nm. Then, 40 mg of composite film was added to 4 mL of the ABTS working solution. All reaction mixtures were incubated in the dark for 30 min. The absorbance of the DPPH reaction system was measured at 517 nm, and the absorbance of the ABTS reaction system was detected at 734 nm. The free radical-scavenging activity was calculated according to Equation (11):

(11) $$\mathrm{Free}\;\mathrm{radical}-\mathrm{scavenging}\;\mathrm{activity}\;\left(\%\right)=\left(1-\frac{A_{1}-A_{2}}{A_{0}}\right)\times 100\%$$

where A_0_ is the absorbance of the blank control (anhydrous ethanol + DPPH working solution for DPPH assay; deionized water + ABTS working solution for ABTS assay, without film sample); A_1_ is the absorbance of the sample (composite film + corresponding radical working solution); and A_2_ is the absorbance of the sample background (negative control, composite film + corresponding solvent, without radical reagent).

#### 2.5.6. Antibacterial Activity Tests

The antibacterial activity of the film specimens was assessed following the experimental procedure published by Qin et al. [26] All films were trimmed into 1 cm diameter round slices and disinfected with UV irradiation over a 1 h period. Four viable bacterial strains, including *S. aureus*, *E. coli*, *P. aeruginosa*, and *B. thermosphacta*, underwent two rounds of activation and subculture, and then were serially diluted to 10^7^ CFU/mL to prepare microbial suspensions. A volume of 200 μL individual bacterial suspension was uniformly coated onto LB agar (TSA agar was adopted for *B. thermosphacta*) to prepare culture plates. An Oxford cup with an outer diameter of 10 mm was used to punch holes in the plates, and the ultraviolet-sterilized film discs were placed in the holes, with 100 μL of sterile physiological saline added, and incubated at 37 °C (25 °C for *B. thermosphacta*) for 24 h.

The optical density values were determined according to the method reported by Huang et al. [27]. Briefly, 1 mL of bacterial suspension was added to 9 mL of physiological saline, 0.3 g of film was weighed, and then the suspension was added after ultraviolet irradiation for 1 h (the setting without adding the film was regarded as the control group). The sample was incubated in a 37 °C (25 °C for *B. thermosphacta*) incubator for 12 h. Optical density (OD_600_) was detected every 3 h.

### 2.6. Application of Films in Chilled-Beef Preservation

#### 2.6.1. Preparation of Beef Samples Covered with Films

Fresh beef was cleaned with sterile water and dried, and then segmented into 2 cm × 2 cm × 2 cm cubic specimens weighing around 20 g under sterile surroundings. The prepared meat was divided into four experimental groups (control, PE (low-density polyethylene cling film, commercial food-grade cling film obtained from a local supermarket), CK, CK-DENPs 15%), with three biological replicates for each group. Beef samples were separately wrapped with one layer of PE, CK, and CK-DENPs 15% films of identical thickness, while unpackaged untreated beef served as the control group. All meat samples were stored in a refrigerator at 4 °C, and physicochemical determinations were performed on days 0, 3, 6, 9, 12, and 15.

#### 2.6.2. Apparent Quality of Beef

Beef samples were photographed at each stage during storage. After film removal, lightness (L*) and redness (a*) were determined with a Color Quest XE colorimeter (HunterLab, USA). Referring to the evaluation system established by Niu et al. [28], appropriate modifications were made in this study. The sensory quality of beef samples packaged with different composite films was evaluated from five indicators, including color, odor, elasticity, viscosity, and overall acceptability. The sensory evaluation panel consisted of ten postgraduate students majoring in food science. All test samples were randomly coded for blind scoring by panel members. Panel sensory assessment and statistical data processing were conducted on days 0, 3, 6, 9, 12 and 15 of storage. The full grading standards for sensory indicators are listed in the supporting documents. Measurement of weight loss rate adopted the testing scheme proposed by Sutharsan et al. [29] with small revisions. Each beef block was weighed to obtain its starting mass, logged as M_1_. All samples were maintained at 4 °C, and their masses were remeasured and logged as M_2_ once every 3 d. The value of weight loss rate was obtained via Equation (12):

(12) $$Weightloss\;rate\left(\%\right)=\frac{\left(M_{1}-M_{2}\right)}{M_{1}}\times 100$$

where M_1_ refers to the initial weight of beef samples (g), and M_2_ represents the sample weight post-storage (g).

#### 2.6.3. Physicochemical and Spoilage Indicators of Beef

Beef pH was measured with a portable pH meter (pH 818M, Smart Sensor Instrument Co., Ltd., Dongguan, China). Total viable count (TVC) measurement followed the revised procedure proposed by Yong et al. [30]. In detail, 5 g beef samples was homogenized with 45 mL sterile brine using a high-speed homogenizer (FSH-2A, Jingfei Instrument Technology Co., Ltd., Hangzhou, China) at 8000 r/min for 1 min to prepare the bacterial suspension with an initial dilution of 10^−1^. Serial 10-fold dilutions were performed using sterile brine to obtain dilutions ranging from 10^−1^ to 10^−8^. A 200 μL portion of suspension at the appropriate dilution was coated on PCA plates. Incubation was carried out at 37 °C for 48 h. Plates with colony counts between 30 and 300 CFU were selected for bacterial enumeration. TVB-N content was quantified with an automatic Kjeldahl analyzer (Qingdao Jingcheng Instrument & Meter Co., Ltd., Qingdao, China) in accordance with GB 5009.228-2016 [31]. In short, 6 g beef sample was extracted with deionized water and filtered; the filtrate was distilled after adding MgO, followed by titration with standard hydrochloric acid. The TBARS value was determined according to GB 5009.181-2016 [32]. Briefly, 0.15 g beef was extracted with trichloroacetic acid and centrifuged. The supernatant was blended with TBA reagent and heated in boiling water, and absorbance at 532 nm was recorded to quantify malondialdehyde. The contents of total protein, total sulfhydryl groups and carbonyl compounds were determined using matching commercial assay kits supplied by Nanjing Jiancheng Bioengineering Institute, Nanjing, China.

### 2.7. Soil-Burial Disintegration and Weight Loss Test

The soil-burial disintegration and weight loss performance of composite films were evaluated according to the work of Liang et al. [33] with slight modifications. Rectangular fragments (3 cm × 4 cm) were cut from the composite films. Prior to burial, the film specimens were dried at 40 °C to a constant weight to obtain the initial dry weight (W_0_). The pre-dried films were buried in nutrient-abundant black soil at a depth of 5 cm. Incubation was performed at ambient temperature (25 ± 2 °C), and soil moisture was maintained at 20 ± 2%. Soil moisture was monitored gravimetrically every two days. Deionized water was sprayed onto the soil surface to compensate for water evaporation and keep the soil moisture stable within the target range. Three replicate specimens of each film were retrieved at 14 d and 28 d. For sample recovery, all recovered materials including broken film fragments were carefully collected. The samples were gently rinsed with deionized water to remove adhered soil particles, dried at 40 °C to a constant weight, and subsequently weighed (W_1_). Weight loss (WL, %) was calculated via Equation (13):

(13) $$WL\left(\%\right)=\frac{W_{0}-W_{1}}{W_{0}}\times 100$$

where W_0_ refers to the film weight before soil burial, and W_1_ denotes the weight after soil burial.

### 2.8. Statistical Analysis

All data were expressed as mean ± standard deviation (SD) with triplicate measurements. Statistical analyses were performed using SPSS 26.0 (SPSS Inc., Chicago, IL, USA). Normality and homogeneity of variance were checked before significance testing. One-way ANOVA was conducted, followed by Duncan’s multiple range test for group comparisons. Differences at *p* < 0.05 were considered statistically significant.

## 3. Results and Discussion

### 3.1. Characterization of DENPs

**Figure 1:**
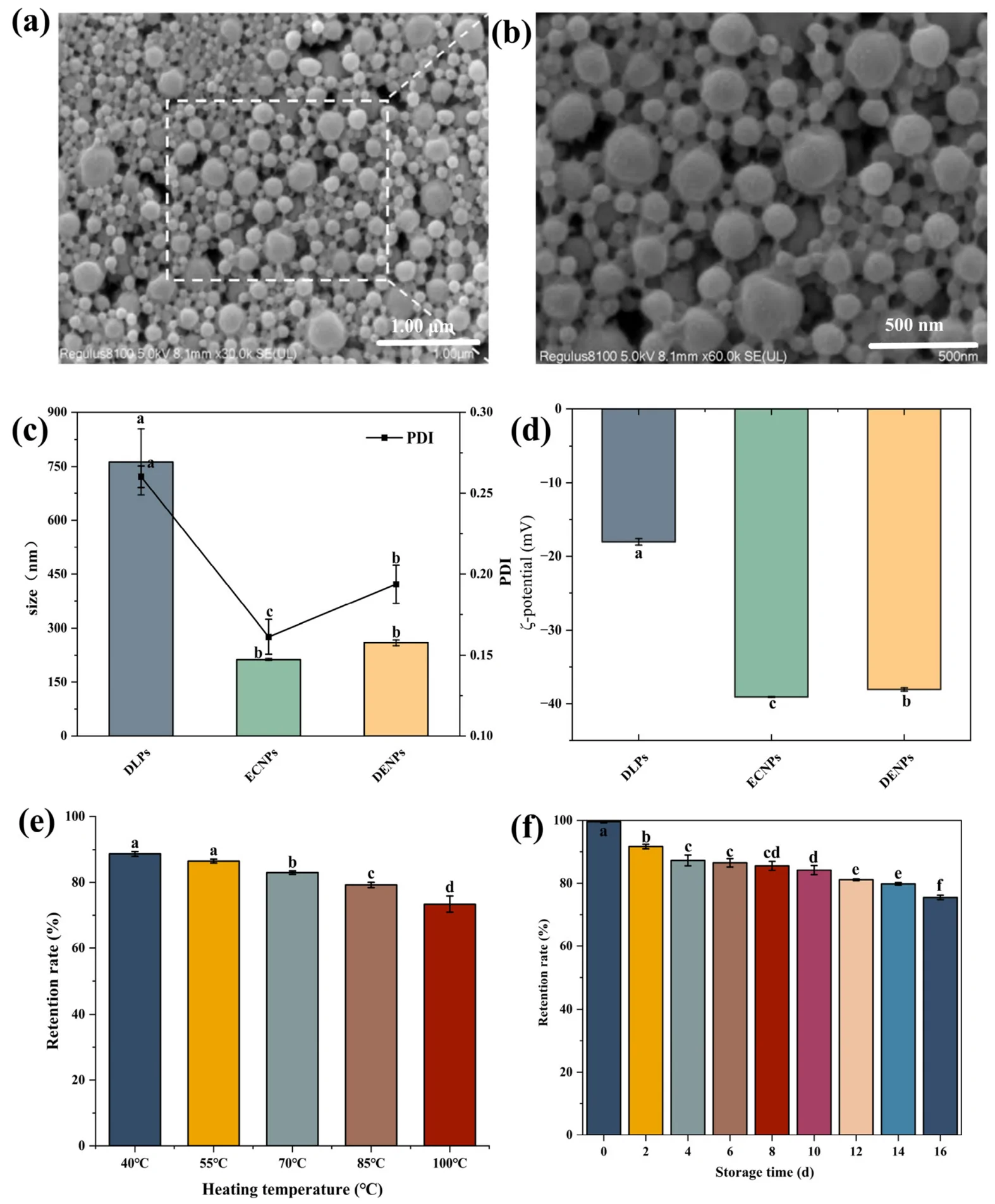
(**a**) SEM images of DENPs, scale bar 1 μm; (**b**) SEM images of DENPs, scale bar 500 nm; (**c**) particle size and polydispersity index (PDI) results of DLPs (D-limonene particles), ECNPs (ethyl cellulose nanoparticles), and DENPs (ethyl cellulose-encapsulated D-limonene nanoparticles); (**d**) zeta potential of DLPs (D-limonene particles), ECNPs (ethyl cellulose nanoparticles), and DENPs (ethyl cellulose-encapsulated D-limonene nanoparticles); (**e**) thermal retention rate of DENPs; (**f**) Storage retention rate of DENPs. Note: Values = mean ± SD, *n* = 3. Lowercase superscripts differ significantly at *p* < 0.05.

As shown in Figure 1a–d, the as-prepared nanoparticles exhibited a regular spherical morphology with uniform particle size distribution. Encapsulation using EC as the wall material effectively reduced the particle size of DLPs. The DENPs had an average particle size of 259.49 ± 8.38 nm, a PDI of 0.194 ± 0.012, and an absolute zeta-potential of 38.05 ± 0.23 mV. These results indicated that encapsulation of D-limonene with EC could improve its dispersibility and stability. The thermal and storage stability results are presented in Figure 1e,f. After water-bath treatment at 100 °C for 60 min, the thermal retention rate of D-limonene reached 79.24%, and the retention rate remained at 75.49% following storage at 4 °C for 16 d. This suggested that ethyl-cellulose-based nano-encapsulation could mitigate the loss of D-limonene under high-temperature and storage conditions to a certain extent and provide partial protection for the core material.

### 3.2. Characterization of Films

#### 3.2.1. Film Appearance and SEM

**Figure 2:**
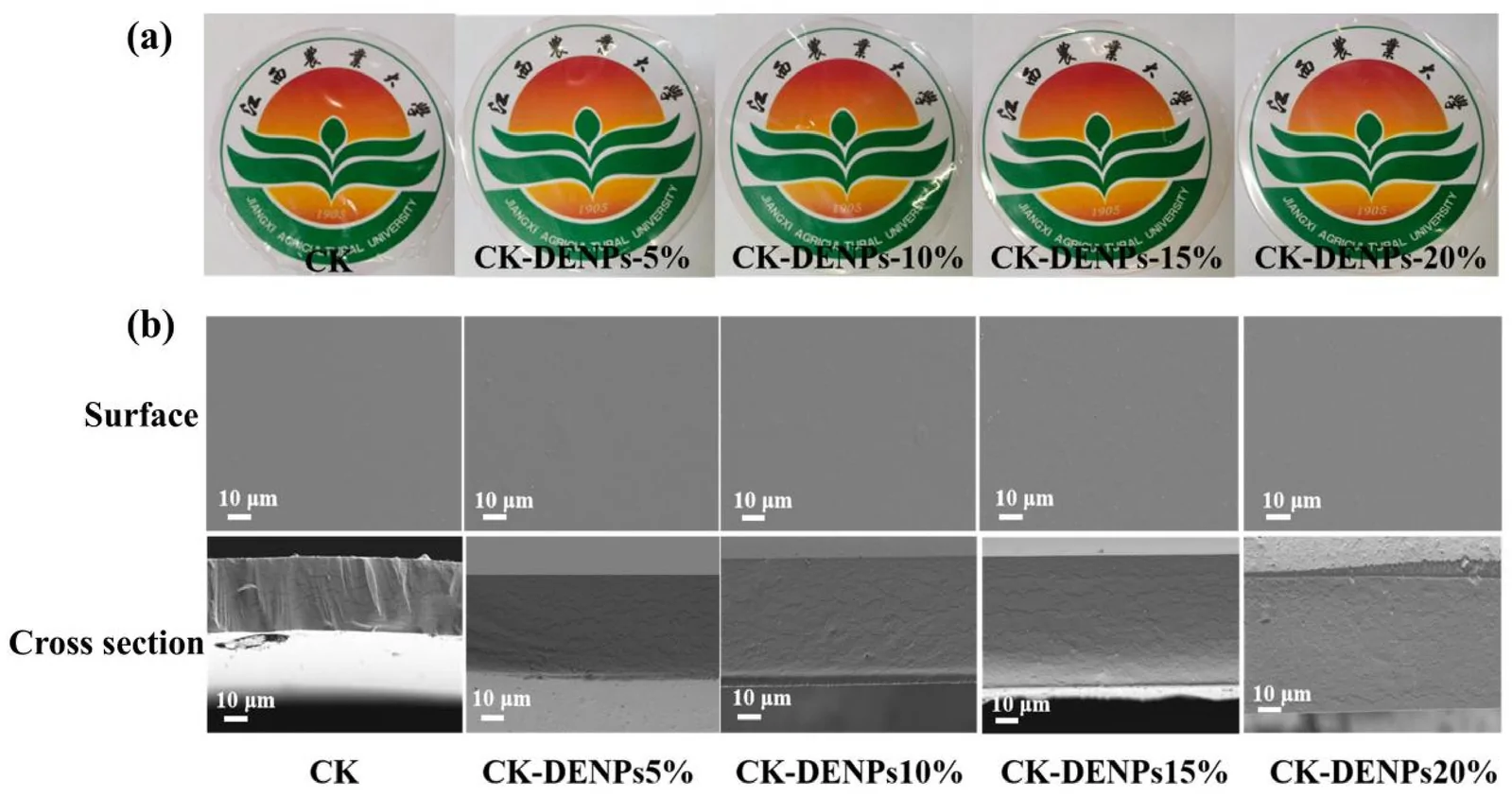
(**a**) Digital photographs of composite films. The Jiangxi Agricultural University emblem was used as the background to highlight the color difference among film samples; (**b**) SEM images displaying surface and cross-sectional morphologies of films (scale bar: 10 μm).

Figure 2a shows the appearance of composite films. All composite films were transparent with smooth even surfaces, free of cracks and pores. SEM micrographs (scale bar:10 μm) in Figure 2b revealed surface and cross-section microstructures. The control film had smooth surfaces and dense texture, demonstrating great compatibility between CS and KGM. At 5–15% DENP loadings, the nanoparticles dispersed homogeneously without aggregation. The films exhibited smooth surfaces and progressively tighter, denser cross-sectional interfaces. These results verified good compatibility between DENPs and the matrix, with no structural damage induced by nanoparticle incorporation. The dense nanoparticle network may strengthen intermolecular interactions, which possibly contributes to the improvement in mechanical and barrier performances of the composite film [34]. When the DENP loading increased to 20%, increased surface roughness and partial nanoparticle agglomeration could be observed for the films. It is supposed that such agglomeration may give rise to stress concentration, which possibly impairs the microstructure of composite films [35,36].

#### 3.2.2. Color and UV–Vis Transmittance Analysis

**Figure 3:**
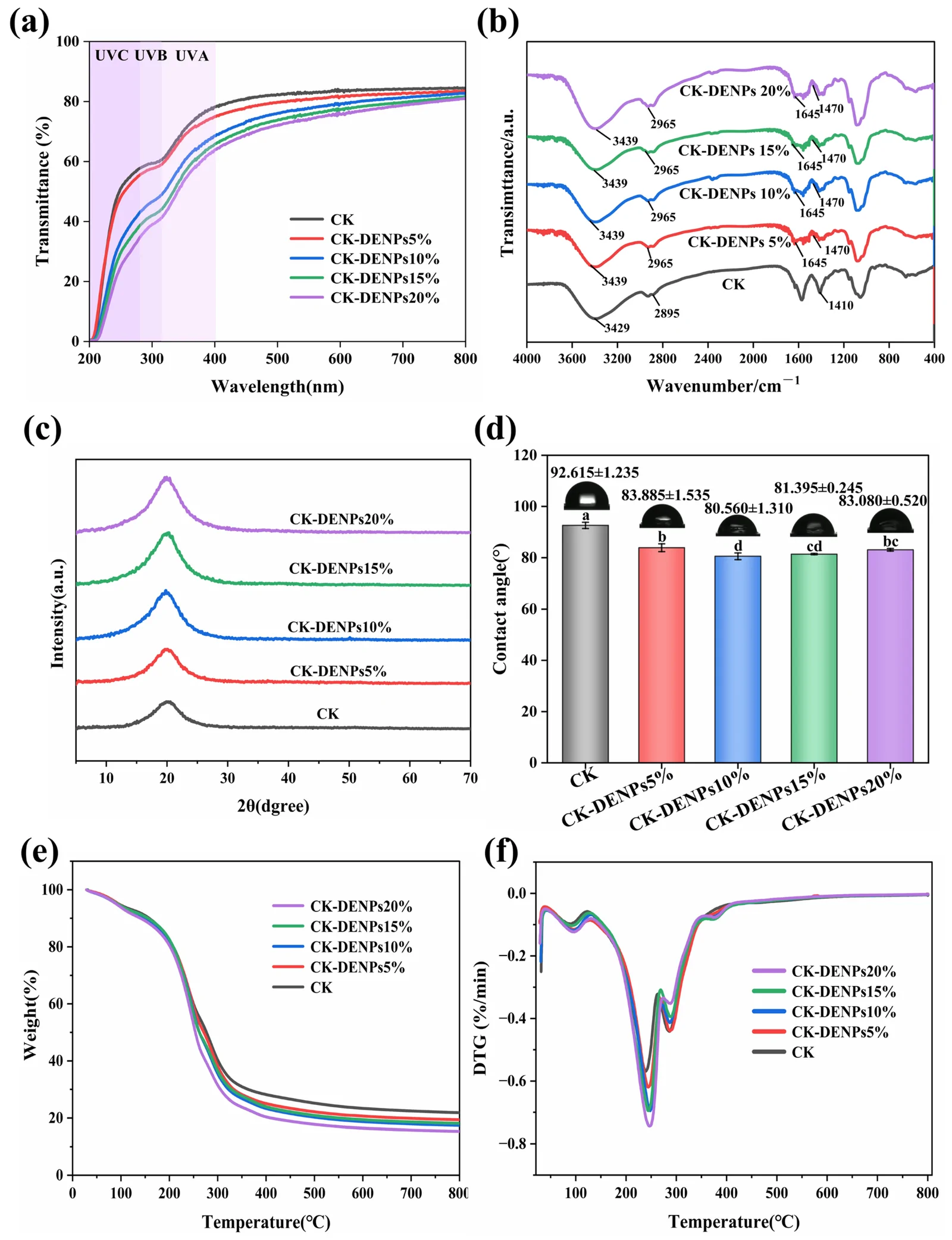
(**a**) UV barrier properties; (**b**) FTIR spectrograms; (**c**) X-ray diffraction profiles; (**d**) water contact angles; (**e**) TG curves; (**f**) DTG curves of composite films. Note: Values = mean ± SD, *n* = 3. Lowercase superscripts differ significantly at *p* < 0.05.

Table 1 illustrates variations in color parameters for all film specimens. Relative to CK films, raising the loading content of DENPs triggered notable reductions in the lightness (L*) and whiteness index (WI) of CK-DENPs composite films. On the contrary, remarkable elevations were detected in the yellowness index (YI) and yellow-blue chromatic coordinate (b*), accompanied by a sustained upward trend in total color difference (ΔE). At a DENP incorporation ratio of 20%, the composite film possessed the maximum total color difference (ΔE = 3.64) and peak yellowness index (YI = 5.92). The results indicated that a high dosage of DENPs endowed the composite film with an obviously yellowish and dark appearance, which may be presumably associated with the inherent color of D-limonene essential oil and the light-scattering effect of DENPs within the film matrix [37]. Figure 3a reveals that nanoparticles improve composite films UV shielding, with performance rising at higher DENP loadings. All films exhibit visible transmittance above 80%, maintaining high transparency while boosting UV resistance. This result was consistent with the color difference analysis and could meet the appearance requirements of food packaging materials.

#### 3.2.3. FTIR and XRD

FTIR spectra collected from all films specimens are displayed in Figure 3b. Compared with the CK film, the CK-DENPs composite film exhibited an obvious blue shift in the characteristic stretching vibration peak of hydroxyl groups (O-H) at 3439 cm^−1^, and a new characteristic absorption peak emerged at 1470 cm^−1^. The O-H characteristic band at 3439 cm^−1^ underwent a blue shift, which may presumably arise from molecular aggregation and the newly formed hydrogen bonding between the blank film substrate and DENPs. The absorbance intensity at 2965 cm^−1^ increased continuously with the rise in DENP content. This band could be assigned to the C-H stretching vibrations of EC [38]. The newly appeared characteristic absorption peak near 1470 cm^−1^ could be assigned to the C-H bending vibrations of aliphatic groups and vibration signals of alkene structures originating from D-limonene essential oil, which may serve as indirect evidence for the incorporation of DENPs within the composite film matrix at the functional-group level [39]. Furthermore, FTIR band shifts, contour variations and intensity changes may indirectly suggest enhanced intermolecular interactions upon DENP incorporation. It could be inferred that the introduced hydrophobic functional groups potentially contribute to the modification in micro-structural features and comprehensive properties of composite films. XRD characterization was performed to explore how incorporated DENPs may influence the crystalline stacking of the film substrate. Figure 3c shows a broad diffraction halo near 2θ ≈ 20° for five film groups, which could be attributed to the mainly amorphous structure of CS, KGM and EC matrices. As the DENP loading increased, the intensity of this broad band around 2θ ≈ 20° gradually rose. We speculate that this intensity change may stem from locally ordered aggregated domains of EC chains formed inside DENPs during D-limonene encapsulation. Higher DENP dosage might increase the fraction of such local ordered domains, which could explain the elevated diffraction intensity [40].

#### 3.2.4. Water Contact Angle

Figure 3d presents the water contact angle (WCA) results of composite films. The CK film exhibited a WCA higher than 90°. The WCA decreased first and then increased with the rise in DENP loading, which may suggest that DENPs could alter the surface molecular arrangement and exposure of hydrophilic groups, instead of merely introducing hydrophobic components. At a low DENP content, more inner hydrophilic groups might migrate toward the film surface to reduce WCA. Excessive DENPs may form surface hydrophobic aggregates, contributing to WCA elevation. This observation shows certain similarities with previous report Li et al. [41].

#### 3.2.5. Mechanical Properties

Tensile strength (TS) and elongation at break (EAB) serve as core evaluation indexes for the mechanical performance of composite films. According to the data in Table 2, film thickness increased steadily with the rise in DENP proportion. Both TS and EAB increased first and then decreased across all groups, reaching the maximum at a DENP dosage of 15%, which was 72% and 67% higher than those of the blank film, respectively. The favorable mechanical performance at moderate DENP addition may result from uniform nanoparticle dispersion, which possibly strengthens intermolecular interactions and optimizes the internal microstructure of the film matrix [42]. In contrast, the reduction in mechanical properties at 20% DENP loading may stem from microstructural defects triggered by nanoparticle agglomeration, which is well supported by the aforementioned SEM morphological results. It is suggested that rational DENP addition contributes to mechanical improvement in the films, whereas excessive nanoparticle incorporation tends to impair film structural integrity and mechanical durability [43].

#### 3.2.6. Water Resistance and Barrier Performances

Moisture content (MC), water solubility (WS) and swelling degree (SD) reflect the water-holding, water-resistant and structural-stability properties of films [44,45]. According to Table 3, the CK film possessed the maximum MC of 22.93 ± 0.04%, and MC declined significantly with increased DENP loading (*p* < 0.05). DENPs may form extra hydrogen bond interactions with hydroxyl groups on film matrix, which could strengthen film networks, reduce hydrophilic adsorption sites, and the densified matrix may retard water penetration and retention [46]. WS decreased markedly upon DENP incorporation (*p* < 0.05). The WS of CK was 17.54 ± 0.28%, and fell to 11.37 ± 0.81% at 20% DENPs, with larger inter-group differences at higher filler levels. SD decreased from 21.47 ± 0.62% (CK) to 5.63 ± 0.40% for the DENPs 20% group. These observations indicate that DENPs could suppress film swelling, showing similar trends to those reported by Zhang et al. [47].

Water vapor permeability (WVP) characterizes the barrier performance of food-packaging films; lower WVP retards moisture exchange and extends food shelf-life [48]. As shown in Table 3, DENPs of 5–10% effectively reduced WVP with the minimum at 10% loading, whereas WVP rose at 20% DENPs. Well-dispersed DENPs may generate hydrogen bond interactions with CK chains to construct denser networks and block water vapor pathways. Nevertheless, excessive DENPs are prone to aggregation, which may create structural voids inside the matrix, as supported by prior SEM results.

Table 3 reveals that oxygen permeability (OP) decreased first and then increased with rising DENP loading, reaching its minimum at 15% dosage. Low OP may restrain oxygen penetration within packages, which could slow food oxidation and the growth of aerobic spoilage microorganisms such as Pseudomonas, and potentially extend food shelf-life [49]. The OP increase observed at 20% DENPs might be related to severe nanoparticle aggregation. This tendency is comparable with previous reports indicating that filler agglomeration can weaken matrix compactness and structural integrity [50].

#### 3.2.7. Thermal Stability

Thermogravimetric (TG) and derivative thermogravimetric (DTG) analyses were used to evaluate the thermal stability of the films, with TG-DTG curves shown in Figure 3e,f. The minor mass loss below 150 °C may be attributed to the release of bound water and a volatile essential oil [51]. Major thermal degradation at 150–400 °C may correspond to chain scission and carbonization of CS, KGM and EC. Higher DENP loading seems to accelerate mass loss. It is supposed that encapsulated D-limonene may decompose along with the matrix, possibly reducing residue yield and thermal stability [52]. The main DTG peaks at 200–350 °C intensify with increasing DENP content. Combined with TG data, this observation may suggest potential synergistic degradation between the essential oil and substrate, which could lower thermal resistance [53].

#### 3.2.8. Antioxidant Activity

**Figure 4:**
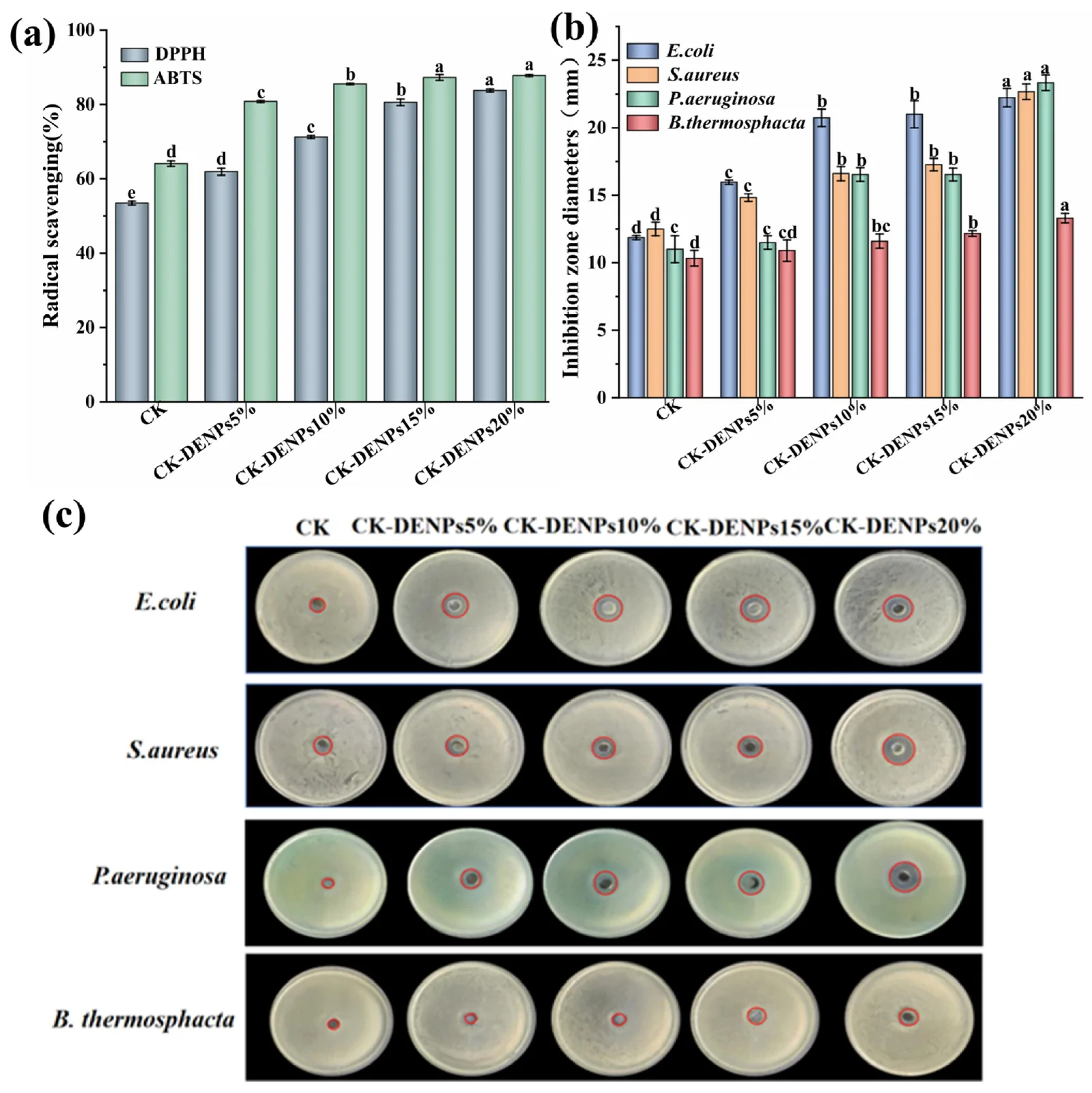
(**a**) DPPH and ABTS free radical-scavenging capacities; (**b**) diameter of inhibition zone against *E. coli*, *S. aureus*, *P. aeruginosa*, and *B. thermosphacta*; (**c**) inhibition zones against *E. coli*, *S. aureus*, *P. aeruginosa* and *B. thermosphacta*. Note: Values = mean ± SD, *n* = 3. Lowercase superscripts differ significantly at *p* < 0.05.

Figure 4a presents the antioxidant activity of composite films. The CK film exhibited a certain level of DPPH and ABTS radical-scavenging capacity, and both scavenging activities increased gradually with the rise in DENP loading. The blank film showed inherent radical-scavenging ability, while the incorporation of DENPs further boosted the antioxidant performance of composite films [54]. The improved antioxidant performance is mainly derived from D-limonene encapsulated within DENPs, which acts as the primary radical-scavenging component contributed by the nanoparticles in this system. Ethyl-cellulose-based nanoparticles serve as protective carriers and mitigate the high volatility of D-limonene; the encapsulated active substance can be gradually released from the film matrix and interact with free-radical species to improve scavenging efficiency. The antioxidant enhancement follows an obvious dose-dependent effect at lower DENP concentrations. Notably, no significant difference was observed between the 15% and 20% DENP groups, indicating that the radical-scavenging capacity reached a plateau when DENP loading exceeded 15%. Moreover, the intermolecular interaction between nanoparticles and the film matrix influences the accessibility of D-limonene to free radicals. Excessively strong interfacial bonding may restrict the diffusion of D-limonene, while well-dispersed DENPs within the polymer network favor the contact between bioactive molecules and free radicals. This tendency is comparable with previously reported findings [55,56].

#### 3.2.9. Antibacterial Activity

Figure 4b,c displays antimicrobial inhibitory regions for *E. coli*, *S. aureus*, *P. aeruginosa* and *B. thermosphacta*. The CK film formed minimal inhibition zones, possibly due to chitosan’s cationic antibacterial property. A higher DENP content enlarged the inhibition diameters for all strains, which may result from synergistic bacteriostasis of chitosan and D-limonene. CS might disrupt bacterial membranes electrostatically, while D-limonene exerts its antibacterial effect by acting on bacterial membranes and interfering with bacterial metabolism. Zhang et al. [57] reported that essential-oil-loaded active packaging could achieve pH-triggered release of bioactive components during meat storage. The released essential oils disrupted bacterial membrane integrity, triggered cytoplasmic leakage and inhibited bacterial proliferation, which is consistent with our proposed bacteriostatic mechanism of D-limonene.

**Figure 5:**
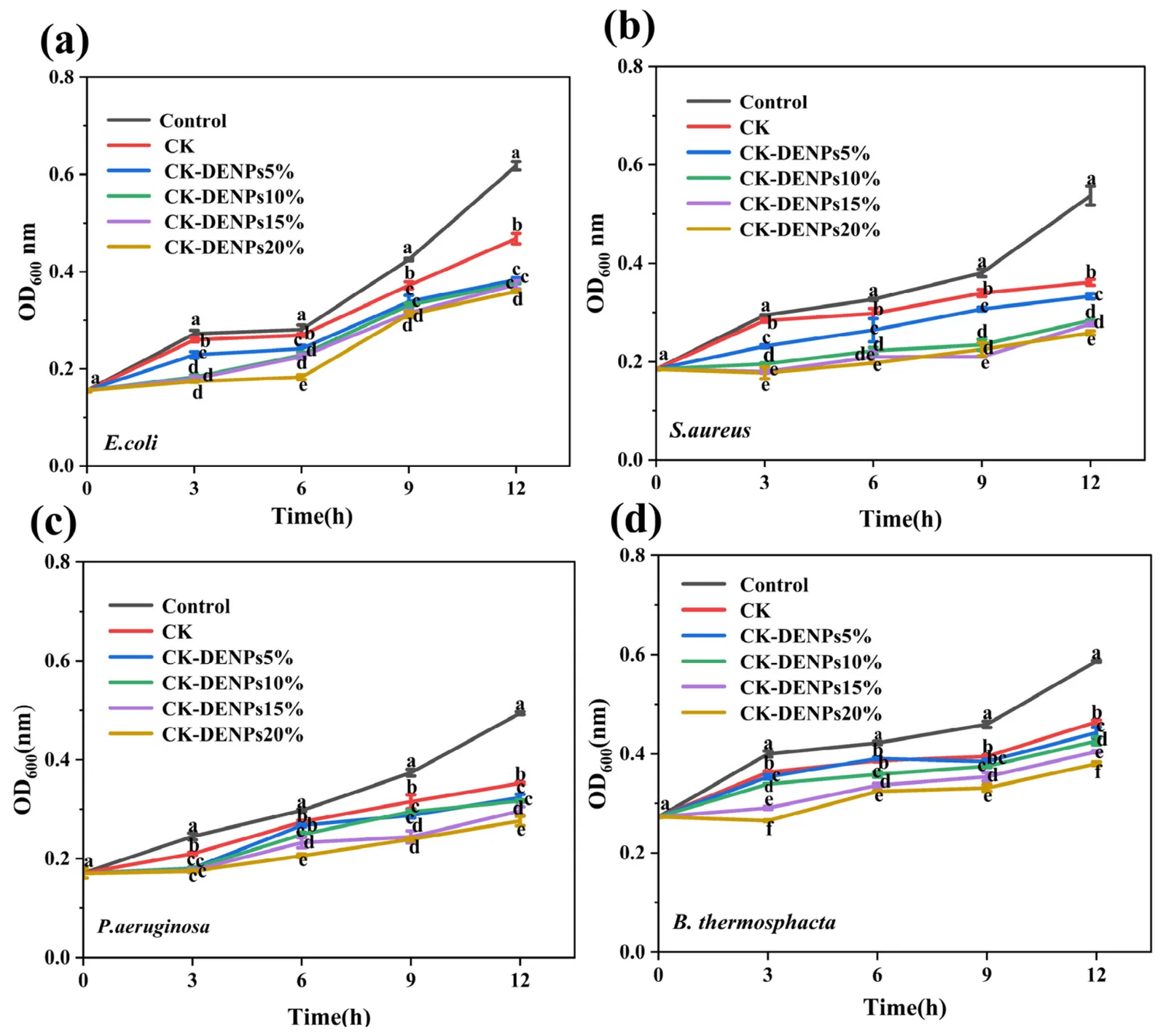
(**a**) Growth curve of *E. coli* after film treatment; (**b**) growth curve of *S. aureus* after film treatment; (**c**) growth curve of *P. aeruginosa* after film treatment; (**d**) growth curves of *B. thermosphacta* after film treatment of the composite films. Note: Values = mean ± SD, *n* = 3. Lowercase superscripts differ significantly at *p* < 0.05.

According to Figure 5a–d, the antibacterial properties of films were evaluated using bacterial OD_600_ growth curves. OD_600_ rose throughout incubation, whereas films with a higher DENP content displayed markedly lower absorbance (*p* < 0.05), which might restrain bacterial multiplication and metabolism. The results agreed with inhibition zone data, revealing the composite films served as promising antibacterial packaging materials.

### 3.3. Effect of the Films on the Preservation Performance of Chilled Beef

#### 3.3.1. Visual Appearance and Sensory Attributes of Beef

**Figure 6:**
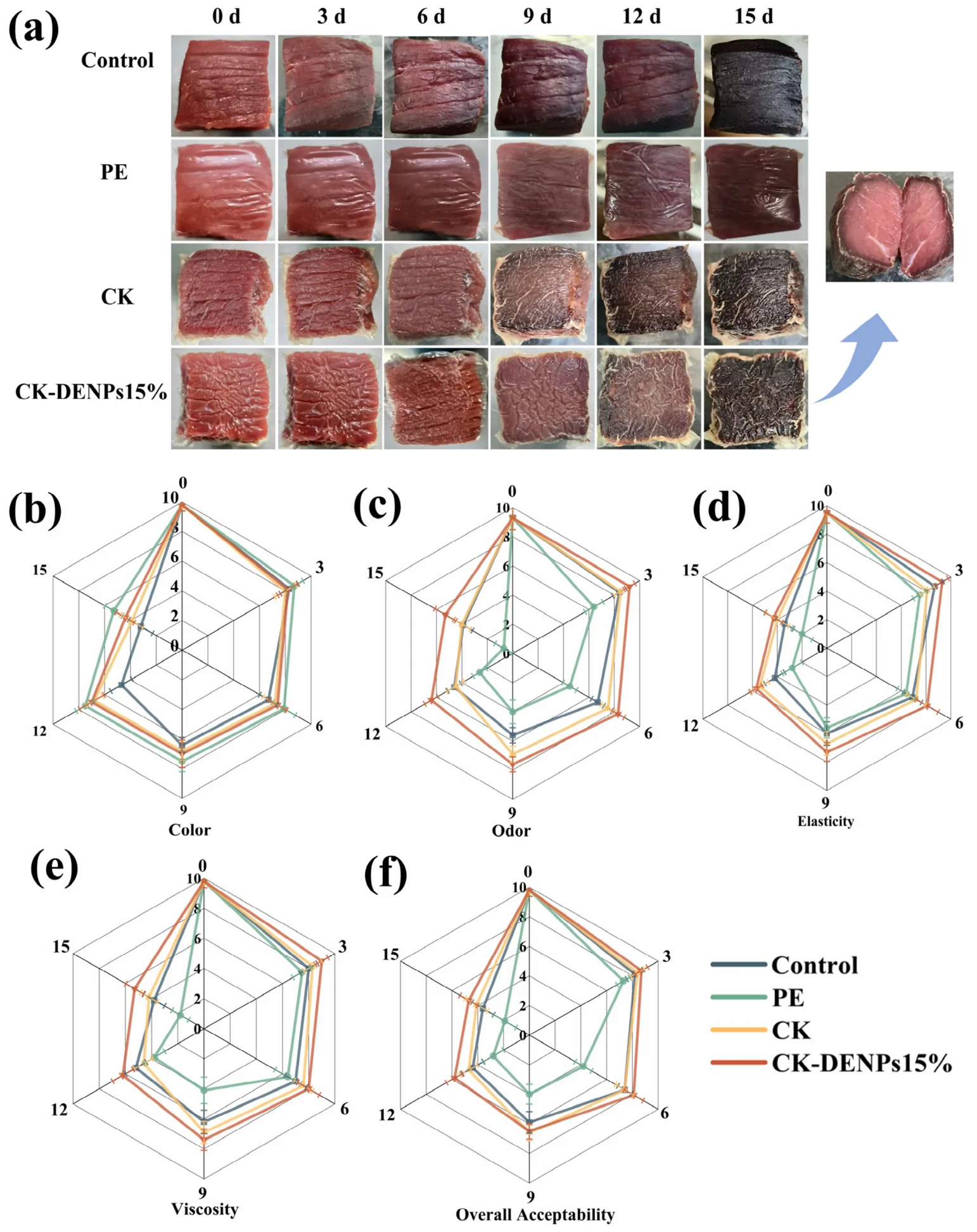
(**a**) Changes in appearance; (**b**–**f**) sensory evaluation ((**b**): color, (**c**): odor, (**d**): elasticity, (**e**): viscosity, (**f**): overall acceptability). Note: Values = mean ± SD, *n* = 3.

Visual appearance can partly reflect the water retention, color-protective and fresh-keeping effects of packaging systems on beef samples. As shown in Figure 6a, the control beef exhibited serious water loss and apparent browning, which may result from myoglobin oxidation. The PE film may maintain beef moisture and color only through physical barrier effects without antibacterial property, which might lead to spoilage and browning during later storage. The pure CK film showed limited preservation capacity with obvious color deterioration. It is presumed that the compact matrix of the CK-DENPs 15% film may provide physical barrier, while DENPs may exert antioxidant and antibacterial effects. This composite film could retard oxygen penetration and water loss, delay myoglobin oxidation and microbial growth, and thus help maintain the bright-red, moist appearance of beef over storage [58]. Sensory evaluation results in Figure 6b–f further support the above trend. The PE film achieved relatively high color scores owing to its favorable oxygen-barrier property, while CK-DENPs 15% showed better performance in odor, elasticity and overall acceptability. It is presumed that DENPs may suppress protein and lipid oxidation, which could reduce the formation of off-flavors [28].

#### 3.3.2. Physicochemical Quality of Beef

**Figure 7:**
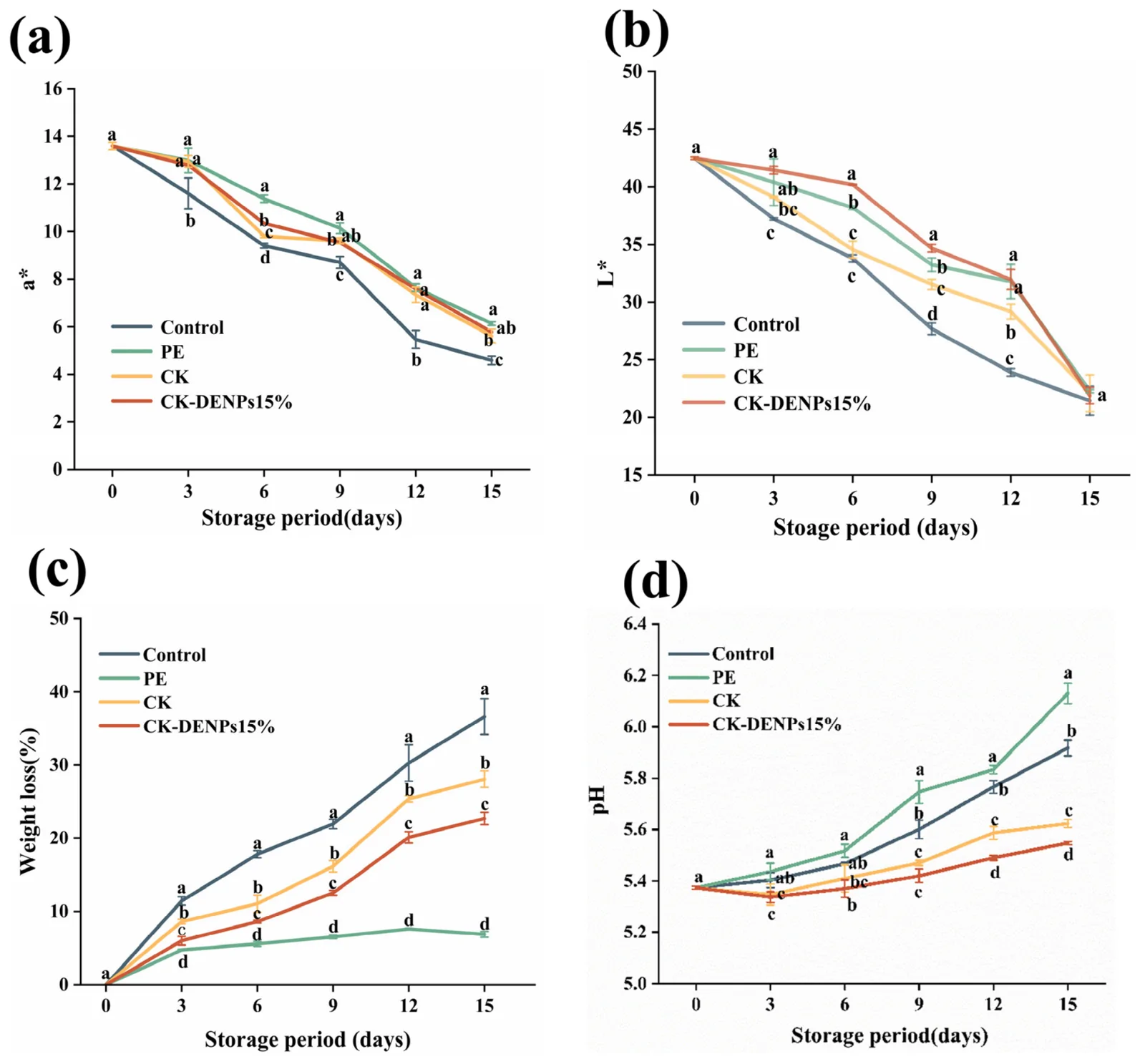
(**a**) a* value; (**b**) L* value; (**c**) weight loss rate of beef during storage; (**d**) pH value of beef during storage. Note: Values = mean ± SD, *n* = 3. Lowercase superscripts differ significantly at *p* < 0.05.

Color parameters a* and L* further quantified color changes, as shown in Figure 7a,b. The a* and L* values of all groups decreased significantly with storage time, which is in accordance with visual observations. It is supposed that the relatively low oxygen permeability of the PE film may account for its slower decline in a*. By comparison, the CK-DENPs 15% group exhibited the mildest L* reduction. This phenomenon may be related to the inhibition of oxidation and water loss by bioactive DENPs, which is consistent with previous reports on color retention under low-oxygen conditions [59,60].

As shown in Figure 7c, the weight loss rate of all film-packaged samples increased markedly during storage, and the control group showed the fastest water loss. The PE film exhibited better moisture barrier properties with the lowest weight loss value. Compared with the CK film, CK-DENPs 15% significantly reduced weight loss (*p* < 0.05). This may be related to its denser microstructure, which could hinder internal water migration. In short, the compact film matrix may provide physical barrier effects, and DENPs may contribute bioactive functions. Their combined effects might help the CK-DENPs 15% composite film maintain beef color and water-holding capacity, and inhibit spoilage to some extent during storage.

pH may serve as an indicator for microbial metabolism and spoilage level of beef. As shown in Figure 7d, the pH values of all samples slightly decreased in the early storage stage, which may relate to lactic acid formation from glycogen glycolysis. A marked pH rise was observed at later storage, possibly due to alkaline metabolites produced when microbes degrade nitrogen-containing substrates. This biphasic pH change trend is consistent with previous observations on chilled meat packaged with bio-active films. Liang et al. [61] retarded the pH increase by relying on the synergistic antibacterial activity of ZIF-8 and gallic acid, whereas Lin et al. [62] realized such an effect by incorporating a lemon essential oil nanoemulsion into a polysaccharide matrix. Our system achieves comparable inhibitory effects against pH elevation through the combined action of the matrix physical barrier and bioactive DENPs. For CK-DENPs 15%, the combination of matrix physical barrier and bioactive DENPs may slow the pH increase and lower the accumulation of alkaline metabolites [63].

#### 3.3.3. Spoilage-Related Indices of Beef

**Figure 8:**
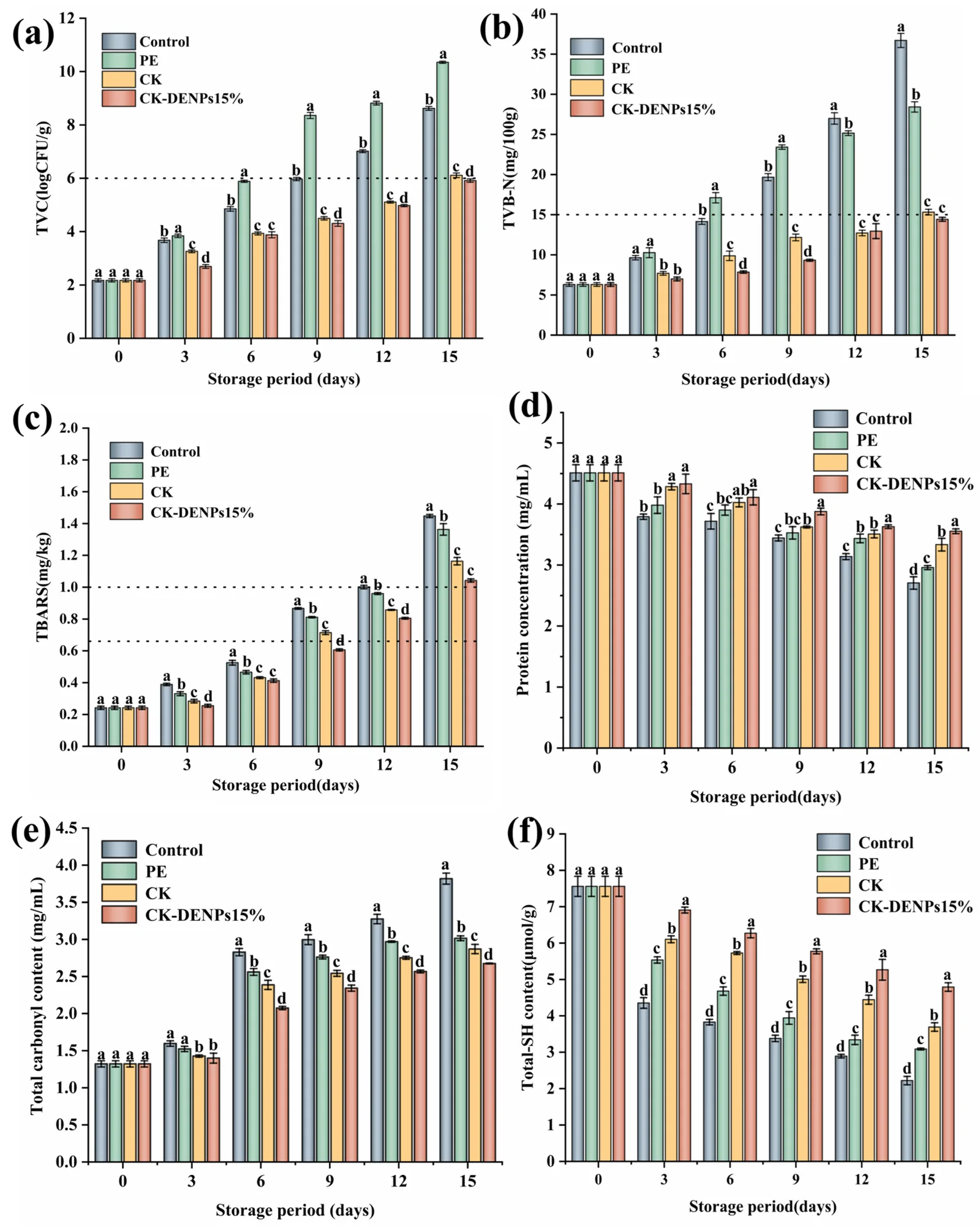
Changes in (**a**) TVC; (**b**) TVB-N; (**c**) TBARS; (**d**) protein concentration; (**e**) total carbonyl content; (**f**) total -SH content. Note: Values = mean ± SD, *n* = 3. Lowercase superscripts differ significantly at *p* < 0.05. The dotted line in (**a**) is the TVC freshness threshold of beef (6 log CFU/g); the dotted line in (**b**) is the TVB-N limit specified in GB 2707 (15 mg/100 g); the two dotted lines in (**c**) correspond to the secondary-quality threshold (0.66 mg MDA/kg) and spoilage threshold (1 mg MDA/kg) of beef based on TBARS value, respectively.

Total viable count (TVC) further verified the differences in microbial contamination and spoilage progression among samples. As shown in Figure 8a, the control and PE groups approached the microbial safety limit on day 9 and day 6, respectively, while the CK group slightly exceeded the limit on day 15. Notably, the CK-DENPs 15% group maintained TVC values within the safe range throughout the storage period. The composite film exerted a physical barrier effect, and the loaded DENPs provided long-term antibacterial activity, thereby significantly extending the microbial safety shelf life of beef [64]. Consistent with the TVC variation trend, total volatile basic nitrogen (TVB-N) was applied to quantitatively evaluate protein spoilage. As displayed in Figure 8b, the PE and control groups successively exceeded the safety threshold and suffered severe spoilage, and the CK group marginally exceeded the standard on day 15. In contrast, the CK-DENPs 15% group remained within the acceptable limit after 15 days of storage. The low oxygen permeability of the composite film reduced the internal oxygen partial pressure, inhibited the metabolism of aerobic spoilage bacteria, decreased the generation of alkaline nitrogenous substances, and ultimately retarded protein spoilage [65].

TBARS were determined to assess lipid oxidation in chilled beef. As displayed in Figure 8c, the TBARS values increased progressively across all groups during refrigerated storage. Compared with the control, DENP-loaded films markedly inhibited TBARS accumulation, indicating that D-limonene containing nanoparticles retarded lipid oxidation, consistent with prior meat preservation studies on active films. Zhang et al. [66] demonstrated that chitosan coatings with a nano-encapsulated essential oil effectively slowed lipid peroxidation of raw pork chops. Notably, the DENP-treated group surpassed the sensory TBARS threshold of 1.0 mg MDA/kg on day 15, even though TVC and TVB-N remained within acceptable ranges. As Cao et al. [64] reported, nanoparticle-reinforced edible films delay but cannot fully prevent beef lipid oxidation. Lipid oxidation and microbial spoilage are partly independent deterioration pathways in muscle foods. Cold storage triggers persistent lipid peroxidation driven by endogenous pro-oxidants such as myoglobin and muscle enzymes. Furthermore, the composite film cannot provide total oxygen exclusion, and released D-limonene is gradually depleted, lowering antioxidant performance in late storage. Hence, despite the prominent antioxidant capacity of the CK-DENPs film, complete suppression of lipid oxidation was not attained by the end of storage.

Besides macroscopic spoilage and oxidation indexes, protein solubility, total carbonyl content and total sulfhydryl content are important parameters for assessing the oxidative degradation of beef myofibrillar proteins. As shown in Figure 8d–f, soluble protein and total -SH contents decreased significantly, whereas total carbonyl content increased obviously in all groups during storage (*p* < 0.05), and obvious differences existed among groups. The control and PE groups exhibited the fastest rates of protein oxidation and degradation. It is supposed that protein oxidation and denaturation may result from massive microbial proliferation, which could cause the loss of soluble protein and active -SH groups. Enhanced protein oxidative modification may further promote carbonyl accumulation [65,67]. The consistent variation trends of the three parameters may suggest that CK-DENPs films could stabilize the protein structure of chilled beef by restraining protein hydrolysis and oxidation simultaneously. Compared with the above two groups, the pure CK film slightly delayed protein deterioration but showed limited protective effects. As regards the CK-DENPs 15% film, it may provide a physical barrier against oxygen and water permeation, while the incorporated DENPs may exhibit free-radical-scavenging and antibacterial activities. Such dual-action effects may reduce hydrolytic loss and maintain soluble protein and -SH groups, and may also suppress side-chain oxidative modification and excessive carbonyl formation [68,69].

### 3.4. Analysis of Film Disintegration Behavior in Soil

**Figure 9:**
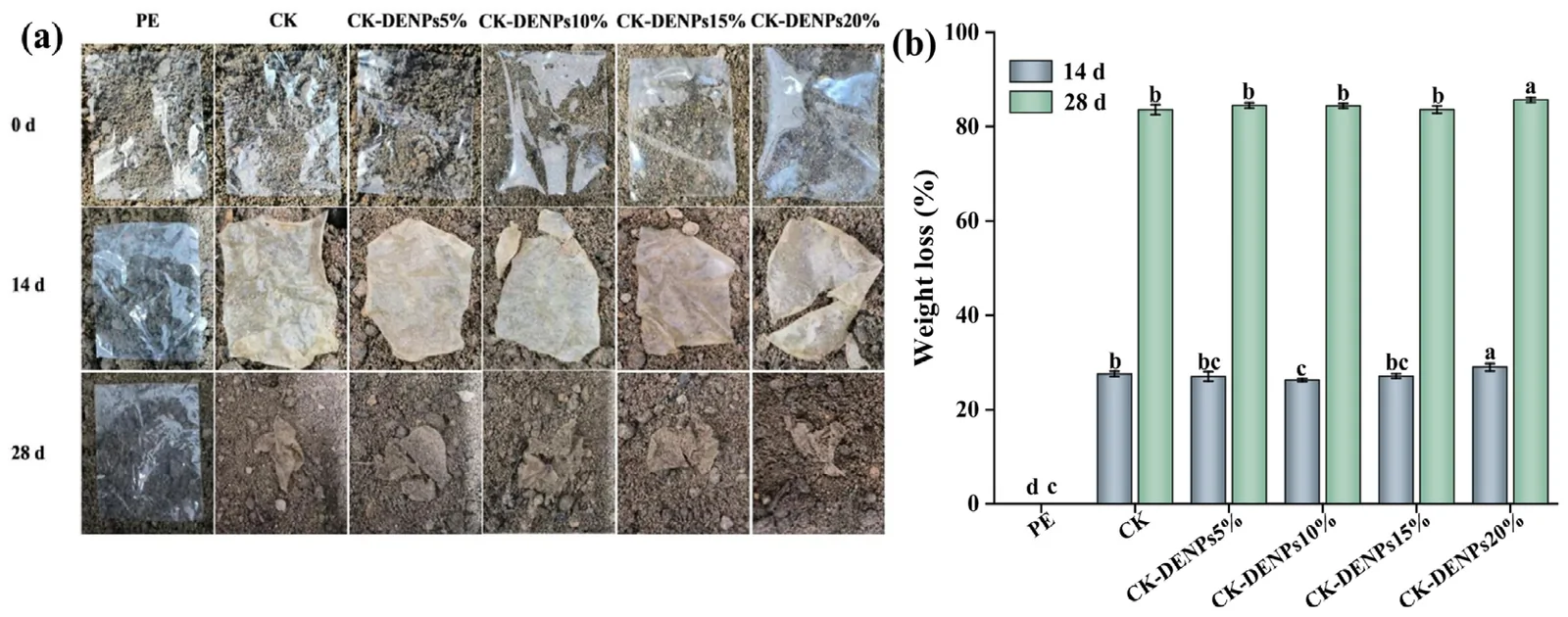
(**a**) Morphologies of films after 14 and 28 d soil disintegration. (**b**) weight loss rates of films at 14 and 28 d. Note: Values = mean ± SD, *n* = 3. Lowercase superscripts differ significantly at *p* < 0.05.

Soil-burial tests were conducted to assess the soil-induced disintegration behavior of films. Film morphologies after 14 and 28 d of soil exposure are shown in Figure 9a, and corresponding weight loss rates are presented in Figure 9b. Chemically inert PE films barely changed within 28 d and persisted long-term in soil. In contrast, all composite films displayed evident brittleness and structural damage at day 14 and nearly full disintegration at day 28, with weight loss rates above 80%. The CK-DENPs 20% film attained the maximum weight loss rate of 85.66 ± 0.52%. Film breakdown mainly proceeds via hydrolysis, microbial enzymatic cleavage and oxidation. The incorporation of DENPs may modulate film microstructure and matrix bioavailability, which could improve soil breakdown efficiency [70]. It is noteworthy that many functional bio-based composite films suffer from reduced soil disintegration performance after modification. For instance, antibacterial Se-loaded starch composite films and cross-linked lignin nanocellulose films exhibited slower soil disintegration because nanofillers suppressed microbial activity or cross-linkers densified polymer networks [71,72]. By contrast, our DENP-incorporated composites still retained high weight loss efficiency (>80%), implying DENPs do not severely inhibit soil microbes. Therefore, the obtained films achieve a desirable trade-off between functional performance and environmental disintegration behavior. Collectively, the as-prepared films combine favorable service stability and good soil disintegration capacity. Due to their relatively rapid breakdown performance in soil, the composite films may be alternatives to conventional PE packaging. They could meet the requirements for short term food preservation, which is favorable for reducing microplastic-related risks and conforms to the development trend of sustainable food packaging.

## 4. Conclusions

In this study, DENPs were first prepared via an antisolvent precipitation method. Functional composite films were further fabricated via solution casting using CS/KGM as the film-forming substrate, and the as-prepared DENPs were incorporated into the film matrix as functional modifiers. The experimental data showed that the addition of these nanoparticles obviously improved tensile properties, UV-shielding ability, and the water- and oxygen-barrier performance of the films, and endowed composite films with favorable antibacterial and antioxidant activities. Structural characterization demonstrated that the incorporation of DENPs altered the microscopic morphology and structural features of the film matrix, which might be responsible for the enhanced structural stability of composite films. Meanwhile, the composite films exhibited favorable soil disintegration performance, which meets the demands for eco-friendly packaging materials. Chilled-beef preservation assays demonstrated that the composite films were capable of inhibiting microbial proliferation and retarding lipid and protein oxidation while preserving the sensory and physicochemical properties of beef. Under the present experimental conditions, these films could extend the acceptable storage period of chilled beef to approximately 15 d. Nevertheless, this study still has some limitations. The preservation evaluation was only conducted under laboratory-simulated cold-storage conditions, and its practical performance in complex commercial cold-chain environments requires further validation. Future work will focus on balancing the sustained release of D-limonene and the long-term storage stability of the films to improve their practical applicability. Moreover, the preservation efficacy of this film system for other perishable meat products warrants further exploration. Overall, the developed CS/KGM-based composite films represent promising eco-friendly active packaging candidates for chilled-meat preservation and may offer insights for designing novel biodegradable food packaging materials.

## Figures and Tables

**Table 1:** Chromatic indicators (L*, a*, b*, ΔE), whiteness index (WI) and yellowness index (YI) for diverse film.

Films	L*	a*	b*	ΔE	WI	YI
CK	93.93 ± 0.01 ^a^	−1.15 ± 0.01 ^a^	2.02 ± 0.05 ^d^	1.61 ± 0.04 ^d^	93.50 ± 0.02 ^a^	3.07 ± 0.07 ^d^
CK-DENPs 5%	93.72 ± 0.04 ^b^	−1.21 ± 0.04 ^b^	2.34 ± 0.19 ^c^	1.99 ± 0.19 ^c^	93.19 ± 0.10 ^b^	3.57 ± 0.29 ^c^
CK-DENPs 10%	93.35 ± 0.16 ^c^	−1.30 ± 0.02 ^c^	3.16 ± 0.17 ^b^	2.89 ± 0.22 ^b^	92.53 ± 0.21 ^c^	4.83 ± 0.27 ^b^
CK-DENPs 15%	93.20 ± 0.06 ^d^	−1.36 ± 0.03 ^d^	3.32 ± 0.11 ^b^	3.11 ± 0.12 ^b^	92.31 ± 0.10 ^c^	5.09 ± 0.17 ^b^
CK-DENPs 20%	93.13 ± 0.05 ^d^	−1.44 ± 0.03 ^e^	3.86 ± 0.27 ^a^	3.64 ± 0.26 ^a^	91.99 ± 0.16 ^d^	5.92 ± 0.42 ^a^

Note: Values = mean ± SD, *n* = 3. Lowercase superscripts differ significantly at *p* < 0.05.

**Table 2:** Film thickness, tensile strength and elongation at break.

Films	Thickness (mm)	TS (MPa)	EAB (%)
CK	0.020 ± 0.001 ^b^	10.48 ± 1.44 ^c^	27.88 ± 1.89 ^e^
CK-DENPs 5%	0.026 ± 0.006 ^ab^	12.73 ± 2.28 ^bc^	32.21 ± 0.72 ^d^
CK-DENPs 10%	0.029 ± 0.008 ^ab^	13.81 ± 1.43 ^b^	40.84 ± 0.82 ^b^
CK-DENPs 15%	0.034 ± 0.006 ^a^	18.05 ± 0.81 ^a^	46.65 ± 2.25 ^a^
CK-DENPs 20%	0.039 ± 0.001 ^a^	14.04 ± 0.90 ^b^	36.00 ± 1.75 ^c^

Note: Values = mean ± SD, *n* = 3. Lowercase superscripts differ significantly at *p* < 0.05.

**Table 3:** Water resistance and barrier performances.

Films	MC (%)	WS (%)	SD (%)	WVP (×10^−8^ g·mm/m^2^·s·Pa)	OP (×10^−8^ g·mm/m^2^·s·Pa)
CK	22.93 ± 0.04 ^a^	17.54 ± 0.28 ^a^	21.47 ± 0.62 ^a^	5.14 ± 0.08 ^ab^	7.32 ± 0.24 ^a^
CK-DENPs 5%	17.58 ± 1.21 ^b^	14.45 ± 0.76 ^b^	18.14 ± 0.63 ^b^	4.77 ± 0.20 ^b^	6.02 ± 0.20 ^b^
CK-DENPs 10%	15.87 ± 0.94 ^bc^	13.49 ± 1.17 ^bc^	12.36 ± 1.10 ^c^	4.61 ± 0.20 ^b^	5.89 ± 0.48 ^bc^
CK-DENPs 15%	14.67 ± 1.60 ^c^	12.60 ± 0.75 ^cd^	8.39 ± 0.39 ^d^	4.91 ± 0.32 ^ab^	5.32 ± 0.36 ^c^
CK-DENPs 20%	14.60 ± 1.42 ^c^	11.37 ± 0.81 ^d^	5.63 ± 0.40 ^e^	5.34 ± 0.44 ^a^	6.82 ± 0.33 ^a^

Note: Values = mean ± SD, *n* = 3. Lowercase superscripts differ significantly at *p* < 0.05.

## Data Availability

The original contributions presented in this study are included in this article/Supplementary Materials. Further inquiries can be directed to the corresponding authors.

## Supplementary Materials

The following supporting information can be downloaded at: https://www.mdpi.com/article/10.3390/foods15213874/s1, Table S1: Score table of sensory evaluation.
