# Supplementary material for: Fabrication of Chitosan/Konjac Glucomannan Active Composite Films Loaded with Ethyl-Cellulose-Coated D-Limonene Nanoparticles for Fresh Beef Preservation
Source: Foods. 2026 Oct 29;15(21):3874. doi: 10.3390/foods15213874 (PMC13763139; doi:10.3390/foods15213874)
Supplement: Supplementary file 1 [file foods-15-03874-s001.zip › foods-4594286-supplementary.pdf]

Supplementary Materials:

Table S1. Score table of sensory evaluation.

Score	Index				
	Color	Odor	Elasticity	Viscosity	Overall acceptability
9-10	Glossy surface with uniformly distributed bright red color	Strong typical fresh meat flavor, no off-odor	Tender texture with excellent elasticity; rapid recovery after pressing	Moist surface without stickiness to touch	Excellent quality, fully acceptable
7-8	Dull light red color	Weak fresh meat aroma, absence of off-flavors	Good elasticity with relatively fast spring-back after compression	Slight surface stickiness	Good quality, well acceptable
5-6	Dark red with slight black discoloration	Slight undesirable off-odor detected	Moderate elasticity with slow recovery	Noticeable sticky surface	Moderate quality, marginally acceptable
3-4	Predominantly dark red or obvious black discoloration	Strong unpleasant off-odor dominates	Very poor elasticity; indentation hardly recovers	Severely sticky surface	Poor quality, barely acceptable
1-2	Most areas turn grayish or black	Extremely strong putrid and rancid odor	No elasticity; permanent indentation after pressing	Extremely slimy and sticky surface	Spoiled completely, unacceptable for consumption
